# MAL2-Induced Actin-Based Protrusion Formation is Anti-Oncogenic in Hepatocellular Carcinoma

**DOI:** 10.3390/cancers12020422

**Published:** 2020-02-12

**Authors:** Alfonso López-Coral, Gianna-Jade del Vecchio, Joeffrey J. Chahine, Bhaskar V. Kallakury, Pamela L. Tuma

**Affiliations:** 1 Department of Biology, The Catholic University of America, Washington, DC 20064, USA; alfonso.lopezcoral@nih.gov (A.L.-C.); giannajade.delvecchio@gmail.com (G.-J.d.V.); Joeffrey.J.Chahine@gunet.georgetown.edu (J.J.C.); 2 Department of Pathology, MedStar Georgetown University Hospital, Washington, DC 20007, USA; KALLAKUB@gunet.georgetown.edu

**Keywords:** hepatocellular carcinoma, cholangiocarcinoma, tumor suppressor, MAL2, actin

## Abstract

Recent studies report that the polarity gene myelin and lymphocyte protein 2 (MAL2), is overexpressed in multiple human carcinomas largely at the transcript level. Because chromosome 8q24 amplification (where MAL2 resides) is associated with hepatocellular- and cholangio-carcinomas, we examined MAL2 protein expression in these human carcinoma lesions and adjacent benign tissue using immunohistochemistry. For comparison, we analyzed renal cell carcinomas that are not associated with chromosome 8q24 amplification. Surprisingly, we found that MAL2 protein levels were decreased in the malignant tissues compared to benign in all three carcinomas, suggesting MAL2 expression may be anti-oncogenic. Consistent with this conclusion, we determined that endogenously overexpressed MAL2 in HCC-derived Hep3B cells or exogenously expressed MAL2 in hepatoma-derived Clone 9 cells (that lack endogenous MAL2) promoted actin-based protrusion formation with a reciprocal decrease in invadopodia. MAL2 overexpression also led to decreased cell migration, invasion and proliferation (to a more modest extent) while loss of MAL2 expression reversed the phenotypes. Mutational analysis revealed that a putative Ena/VASP homology 1 recognition site confers the MAL2-phenotype suggesting its role in tumor suppression involves actin remodeling. To reconcile decreased MAL2 protein expression in human carcinomas and its anti-oncogenic phenotypes with increased transcript levels, we propose a transcriptional regulatory model for MAL2 transient overexpression.

## 1. Introduction

Epithelial cells are vital for the success of humans. They line all bodily organs providing a selective barrier between the external and internal worlds. The plasma membrane of a polarized cell is physically continuous, but functionally and compositionally divided into two domains: the apical and basolateral [1]. This surface polarity is required for proper epithelial cell function. How is surface polarity established and maintained? This question is especially important given that more than 80% of human cancers are derived from polarized epithelial cells and because epithelial-derived cancer cells either lose or fail to achieve polarity. Thus, another important question is how does dysregulation of cell polarity contribute to malignant transformation? This question is of particular importance to us given that the dysregulated expression and/or function of a host of proteins that regulate cell polarity are known to contribute to cancer progression [2]. Prominently on that list is MAL2 (myelin and lymphocyte protein 2), a protein we have been studying for over 15 years.

In that time, we have determined that the 19 kDa tetraspanning integral membrane protein, MAL2, is an important regulator of hepatic polarized protein sorting and have shown that it functions in basolateral secretion, basolateral membrane protein delivery and basolateral-to-apical transcytosis [3,4], but what is important to these studies is the finding that MAL2 expression is highly up-regulated up to 100-fold in a host of human cancers, including renal cell carcinoma, mesothelioma, neuroblastoma, cholangiocarcinoma, and cancers of the colon, liver, stomach, breast, ovary and pancreas [5,6,7,8,9,10,11,12,13,14]. High MAL2 expression has also been linked to bad prognosis in patients with colorectal or pancreatic cancer [5,13]. Genomic instability gains in chromosome 8q24 (where MAL2 resides) are associated with several epithelial cell-derived human cancers [15] and likely explains enhanced MAL2 transcript expression in at least a subset of these cancers. Although MAL2 overexpression is likely not an oncogenic driver of tumorigenesis [2], it may contribute to neoplastic transformation by altering protein targeting that leads to dysregulated protein distributions which ultimately results in changes in cell morphology, signaling and migration.

Because we have been investigating MAL2’s biochemical and functional properties in polarized hepatic cells, we chose to assess its potential role in mediating carcinogenesis in hepatocellular carcinoma (HCC). Importantly, this cancer type is associated with chromosome 8q24 amplification [16,17,18]. We started our investigation with a blinded immunohistochemical analysis of MAL2 protein expression levels (rather than just transcript levels as for the majority of the other studies) in human HCC and adjacent benign tissue. For comparison, we examined MAL2 protein levels in two other cancer types: cholangiocarcinoma, another cancer type associated with chromosome 8q24 amplification [19,20], and in renal cell carcinoma, a cancer type not associated with amplified chromosome 8q24. Surprisingly we found that MAL2 protein expression was significantly decreased in all three carcinomas, more consistent with MAL2 function as a tumor suppressor. To better understand these unexpected results, we performed migration, invasion and proliferation assays in three hepatic-derived cell lines: polarized, differentiated WIF-B cells (with endogenous MAL2 expression), hepatocellular carcinoma-(HCC)-derived Hep3B cells (with endogenous MAL2 levels) and hepatoma-derived Clone9 cells (with no MAL2 expression). The approach was straightforward and in three parts: (1) overexpress MAL2 in WIF-B cells; (2) knock down MAL2 in Hep3B HCC cells; and (3) express MAL2 in Clone 9 hepatoma cells. From these studies we determined that MAL2-induced filopodia-like protrusion formation (likely at the expense of invadopodia and or lamellipodia formation) is anti-oncogenic. Mutational analysis further revealed that MAL2 anti-oncogenic properties are selectively mediated via a cytosolic, N-terminal putative recognition site for EVH1 (enabled, VASP, homology1) motifs present in the actin-associated Ena/VASP proteins. These results further indicate that MAL2’s possible role in tumor suppression involves actin remodeling. We propose a model to reconcile our findings of decreased MAL2 protein expression and its anti-oncogenic phenotypes with the findings from others that MAL2 transcripts are overexpressed in epithelial-derived cancers.

## 2. Results

### 2.1. MAL2 Expression is Decreased in Tumors from Human HCC, CC and RCC

**Figure 1:**
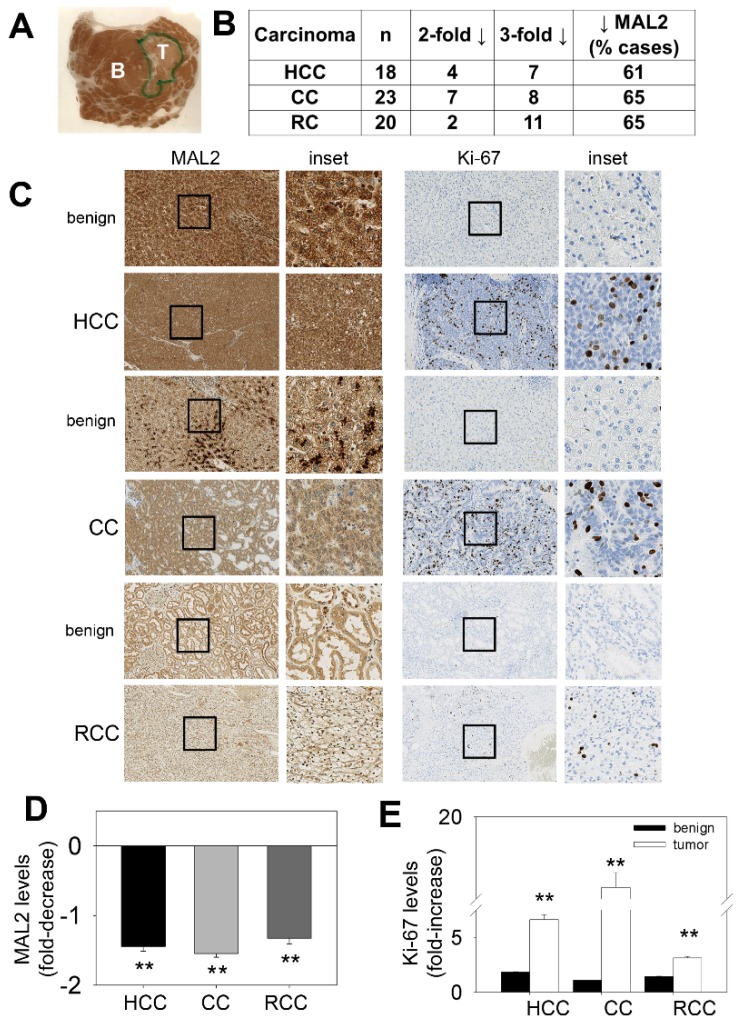
MAL2 expression patterns in human cancer tissue are consistent with its role as a tumor suppressor. (**A**) A liver biopsy was immunostained with diagnostically validated MAL2 antibodies. An unmagnified image of the biopsy mounted on the slide is shown. The areas containing the benign (B) and tumor (T) components are indicated. (**B**) Tumors from hepatocellular carcinoma (HCC), cholangiocarcinoma (CC) and renal cell carcinoma (RCC) biopsies were scored for MAL2 expression. Numbers of patient tumor samples that displayed 2- or 3-fold decreases in MAL2 protein expression are indicated. The total number of cases examined is shown (n) and the percent of cases examined that displayed decreased MAL2 protein expression is indicated. (**C**) Benign and cancerous lesions from human tissues from HCC, CC and RCC as indicated were immunostained with diagnostically validated MAL2 and Ki-67 antibodies. The black boxes indicate the regions of the micrograph enlarged insets. The fold-decrease in MAL2 expression (**D**) and fold-increase in Ki-67 expression (**E**) in cancerous lesions relative to benign across all patient biopsies are plotted. Values represent the mean ± SEM from 18 HCC patients, 23 CC patients and 20 RCC patients. ** *p* ≤ 0.001.

Because of our long-standing interest in defining the mechanisms by which MAL2 regulates polarized hepatic protein sorting, we chose to examine MAL2 expression levels in resected human tissues from HCC and for comparison, from CC (another liver-derived cancer) and RCC using an analytically validated immunohistochemistry (IHC) test for clinical and diagnostic applications. Importantly, both HCC and CC are associated with chromosome 8q24 amplification whereas RCC is not [16,17,18,19,20]. Also importantly, this approach analyzes MAL2 protein levels rather than just transcript levels as was done for the majority of the other studies. In all, 18 different HCC cases, 23 CC cases and 20 RCC cases containing both (adjacent) benign and tumor components were labeled with antibodies against MAL2 and Ki-67 (to assess the proliferative index). To our surprise, MAL2 protein expression was decreased in the malignant tumors even when viewed without magnification. As shown in Figure 1A, the decreased MAL2 labeling was apparent in the tumor component (T) of a patient liver biopsy when examined by eye. Using a blinded semi-quantitative light microscopic scoring method, we further determined that MAL2 expression was significantly down regulated in the majority of samples examined (in 61–65% of the total cases examined) from all three carcinoma types displaying 2- to 3-fold decreases in immunolabeling (Figure 1B).

Examples of the MAL2 staining patterns for each tissue type is shown in Figure 1C. In general and as expected, MAL2 was robustly detected in the terminally differentiated, benign component of all three carcinoma types. When examined at higher magnification (insets), very dense regions of MAL2 labeling were observed. Also as expected, Ki-67 labeling (to mark proliferating cells) was low in the benign component with little to no nuclear staining observed. Also as expected, Ki-67 expression was enhanced in the corresponding tumor lesions with numerous positive nuclei observed. However, MAL2 labeling in the tumor lesions was decreased and no dense immunoreactive clusters were observed. When quantitated across all samples, we determined that MAL2 expression was significantly down-regulated (*p* ≤ 0.001) by approximately two-fold in the tumors (Figure 1D) with a corresponding two- to five-fold increase in Ki-67 labeling (Figure 1E). These results are independently and surprisingly more consistent with MAL2 functioning as a tumor suppressor.

### 2.2. Our Model Systems

**Figure 2:**
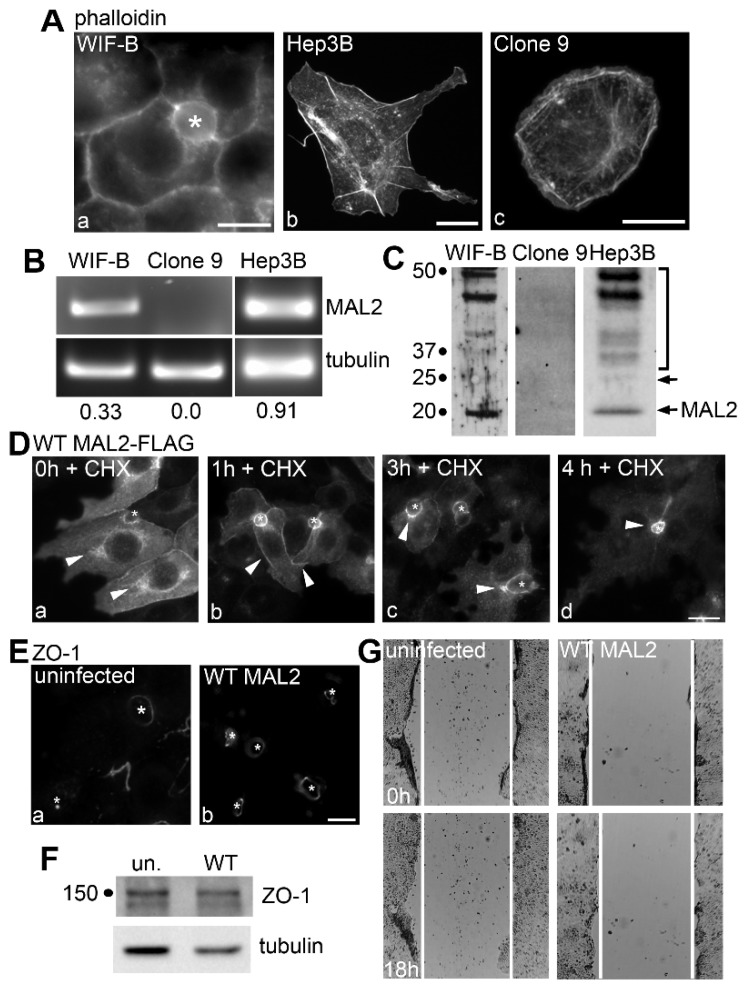
MAL2 is expressed in normal and malignant liver-derived cell lines, but simply overexpressing MAL2 in polarized WIF-B cells is not oncogenic. (**A**) WIF-B (a), Hep3B (b) and Clone 9 cells (c) were labeled for actin with phalloidin. (**B**) Agarose gels are shown of MAL2 (upper panels) and α-tubulin (lower panels) cDNA amplified by RT-PCR from 1 µg total RNA isolated from WIF-B, Clone 9 or Hep3B cells as indicated. Human-specific MAL2 and α-tubulin primers were used for Hep3B cell amplification while rat-specific primers were used for WIF-B and Clone 9 cells. Numbers below the lanes represent the ratio of MAL2 mRNA expression levels normalized to α-tubulin expression levels. (**C**) Lysates from WIF-B and Clone 9 cells were immunoblotted with antibodies specific for rat MAL2 and lysates from Hep3B cells were immunoblotted for human MAL2. Molecular weight standards are indicated on the left in kDa. The bottom arrow marks the predicted 19 kDa MAL2 immunoreactive species. The bracket highlights a diffuse set of bands that has been described by us and others and the upper arrow indicates a 25 kDa species also detected by others. (**D**) WIF-B cells expressing FLAG-tagged wild type (WT) MAL2 were treated with 50 µg/mL of cycloheximide (CHX) for up to 4 h as indicated and immunolabeled for MAL2 with anti-FLAG antibodies. Arrowheads indicate MAL2 localization at the Golgi (a), basolateral membrane (b) SAC (c) or apical surface (d). Asterisks mark bile canaliculi. (**E**) Uninfected WIF-B cells and cells overexpressing wild type MAL2 were immunolabeled for ZO-1. Asterisks indicate bile canaliculi. (**F**) Lysates from uninfected or MAL2-overexpressing cells were immunolabeled for ZO-1 and tubulin as indicated. Molecular weight standards are indicated on the left in kDa. (**G**) Uninfected WIF-B cells or cells overexpressing MAL2 were subjected to a scratch assay. Wound healing was analyzed 0 h (upper panels) and 18 h (lower panels) after the scratch. White lines indicate the edges of the gap. Bar = 10 µm.

To further examine whether MAL2 expression is potentially tumor suppressive, we assayed common oncogenic properties of three hepatic-derived cells: polarized, hepatic WIF-B cells, nonpolarized, HCC-derived Hep3B cells and nonpolarized, hepatoma-derived Clone 9 cells. We first labeled each cell type for filamentous actin with phalloidin to highlight its specific surface features (Figure 2A). WIF-B cells exhibit a typical polarized hepatic morphology with bile canalicular-like structures fully sequestered from the external milieu (marked with an asterisk) with a thick cortical actin web on the cytoplasmic surface of the apical and basolateral plasma membranes (Figure 2A(a)). In contrast, nonpolarized Hep3B cells are characterized by multiple, long, filopodia-like cell-surface protrusions (Figure 2A(b)). Clone 9 cells are also non-polarized, but display a cuboidal morphology with no actin-based protrusions (Figure 2A(c)). Semi-quantitative reverse transcriptase PCR (RT-PCR) confirmed MAL2 mRNA expression in WIF-B and Hep3B cells and the lack of endogenous MAL2 expression in Clone 9 cells (Figure 2B). Immunoblots from whole cell lysates indicated that protein levels mirrored the transcript levels with no endogenous MAL2 expression observed in Clone 9 cells. Because WIF-B cells express rat MAL2 and Hep3B cells express human MAL2, different antibodies were used to probe the lysates such that immunoreactivity cannot be directly compared between immunoblots or with the RT-PCR gels. Nonetheless, MAL2 was detected in both cell lysates (Figure 2C). As previously reported by us and others [4,21], MAL2 immunoreactive species in lysates from WIF-B and Hep3B cells were detected at 19 kDa (the predicted MW), 25 kDa (arrow) and a diffuse set of bands ranging from 30–50 kDa.

### 2.3. MAL2 Overexpression in Polarized WIF-B Cells Is Not Oncogenic

To rule out that MAL2 overexpression alone can promote a loss of cell polarity and a more malignant phenotype, we overexpressed C-terminally FLAG-tagged wild type MAL2 in polarized WIF-B cells using recombinant adenovirus. Because hepatocytes favor the indirect route for the delivery of newly-synthesized apical plasma membrane-spanning proteins to the apical surface [1], it was not surprising to detect the constitutively expressed MAL2 throughout the biosynthetic pipeline and in the intermediates of transcytosis after 24 h of infection. As shown in Figure 2D(a), FLAG-tagged MAL2 was present in the ER (diffuse labeling), the Golgi (discrete, peri-nuclear staining marked with arrows), the basolateral membrane (en route to the apical surface) and at the apical domain itself (Figure 2D(a)). To determine MAL2’s steady state distributions, we treated cells with 50 µg/mL of cycloheximide (CHX) for up to 4 h and monitored the newly-synthesized cohort as it was chased through the biosynthetic pathway. In cells treated for 1 h, the diffuse MAL2 ER labeling and discrete Golgi labeling were lost with a reciprocal increase in MAL2 detection at the basolateral surface (Figure 2D(b)). After 3 h, MAL2 basolateral labeling was lost with increased staining observed just adjacent to the apical surface in the cap-like sub-apical compartment (SAC) (marked with arrows) and at the apical surface itself (Figure 2D(c)). After 4 h, only apical labeling was observed (Figure 2D(d)) confirming that overexpressed MAL2 is apically-located at steady state as for endogenously-expressed MAL2 [4]. This MAL2 “chase” from the ER-to-Golgi biosynthetic pipeline to the basolateral surface, to the SAC and finally to the apical surface further indicate that newly-synthesized MAL2 is delivered to the apical domain via the indirect pathway [22,23,24].

If MAL2 overexpression is oncogenic, a simple prediction is that the polarized WIF-B phenotype should be lost. However, we observed no changes in the distributions (Figure 2E) or protein expression levels (Figure 2F) of the tight junction component, zonula occludens-1 (ZO-1). As shown in Figure 2E, ZO-1 labeling in the belt-like structures surrounding the canalicular cysts at the contact sites of adjacent cells was maintained. When quantitated, no differences in the numbers of bile canaliculi were observed between uninfected (33 bile canaliculi/100 cells) and MAL2 overexpressing cells (39 bile canaliculi/100 cells) further indicating that polarity was not altered by MAL2-overexpression. Another simple prediction is that if MAL2 overexpression is not oncogenic, it should not promote cell migration in scratch assays. Live cell imaging of wound healing confirmed this prediction and revealed no differences in cell migration between control and infected cells. In both cases, no significant wound closure was observed even after 18 h of recovery (Figure 2G). Quantitation of the gap areas confirmed the morphological observations. In both control and wild type MAL2-expressing cells, ~90% of the initial gap area remained (94.0 ± 0.3 and 89.3 ± 2.1%, respectively, *n* = 3) after 18 h of recovery. Together these results indicate that MAL2 overexpression alone is not oncogenic.

### 2.4. MAL2 Knockdown in HCC-Derived Hep3B Cells Decreased Actin-Based Protrusions, yet Enhanced Cell Migration

**Figure 3:**
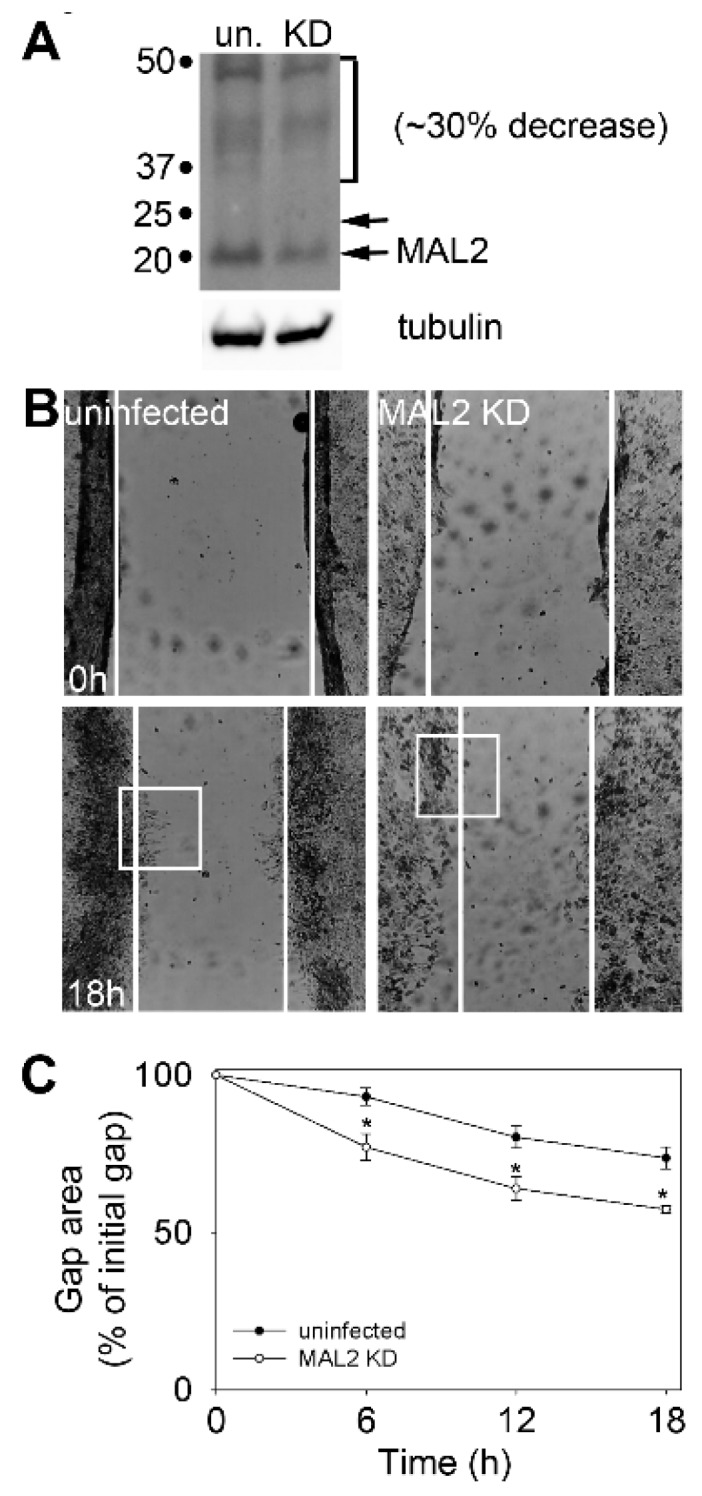
MAL2 knockdown in Hep3B cells enhances cell migration. (**A**) Lysates from uninfected (un) Hep3B cells or cells knocked down (KD) for MAL2 expression with anti-sense MAL2 recombinant adenoviruses for 4 days were immunoblotted for endogenous MAL2 or tubulin. Molecular weight markers are indicated on the left in kDa. A ~30% reduction in MAL2 levels was observed. (**B**) Wound healing was imaged at 0 and 18 h post-scratch. White lines indicate the border of the monolayer along the scratch. Boxed areas were enlarged and shown in Figure 4A. (**C**) The gap area remaining relative to the initial scratch area was determined 0, 6 (*p* ≤ 0.013), 12 (*p* ≤ 0.014) and 18 h (*p* ≤ 0.002) after recovery. The values represent the average ± SEM determined from at least three independent experiments.

To determine whether endogenous MAL2 expression correlates with tumor suppression, we continued our analysis in human HCC-derived Hep3B cells. For these studies we chose an anti-sense approach using recombinant adenovirus as we have described [4,25]. As observed for WIF-B cells [4,25], MAL2 in Hep3B cells is relatively long-lived, and even after 4 days post-infection with anti-sense MAL2 adenoviruses, only partial knockdown (~30% reduction) was achieved (Figure 3A). 

As a read-out for an oncogenic phenotype, we assayed wound closure in control and MAL2 knockdown cells. Control Hep3B cells displayed only modest wound closure after 18 h (Figure 3B, bottom left panel), and when quantitated, only ~20% of the gap was closed (Figure 3C). Hep3B cells with reduced MAL2 expression showed enhanced migration (Figure 3B, bottom right panel) with almost ~30% of the gap closed after 18 h of recovery (Figure 3C). Although modest, this increase in gap closure was significant (*p* ≤ 0.01 for 6 and 12 h and *p* ≤ 0.002 for 18 h) and consistent with the ~30% decrease in MAL2 expression observed.

**Figure 4:**
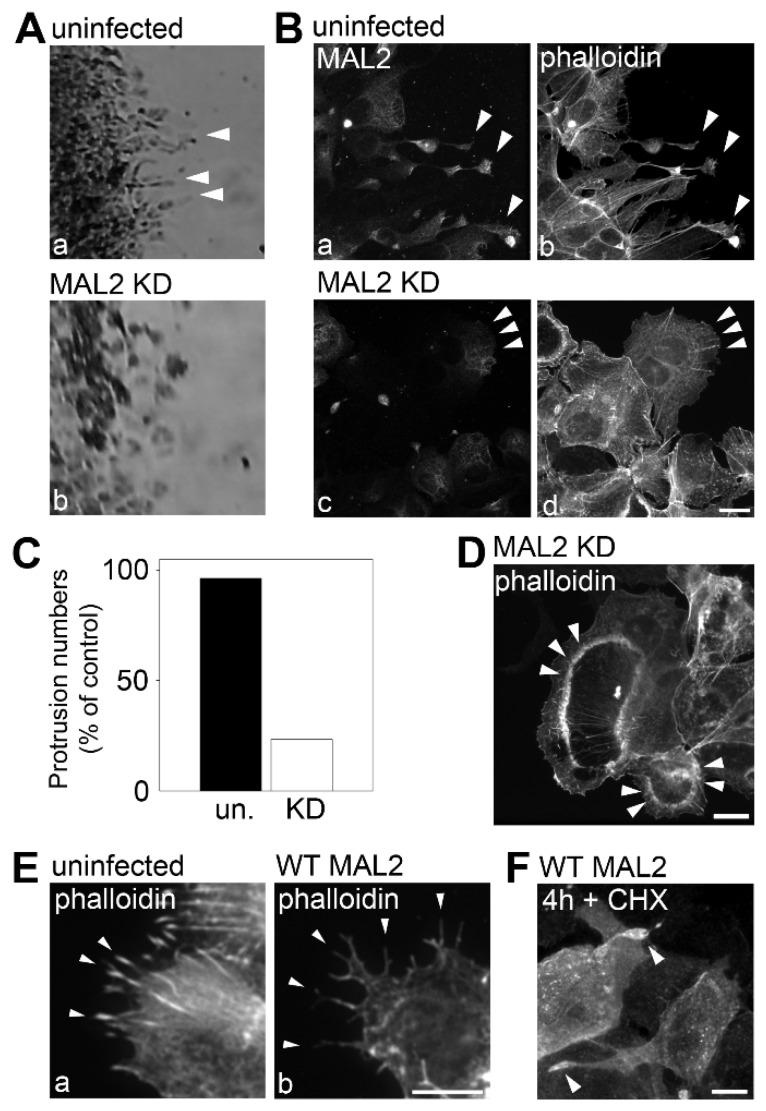
MAL2 expression in Hep3B cells correlates with actin-based protrusion formation. (**A**) The boxed areas from Figure 3B were enlarged. Arrowheads indicate long protrusions present in uninfected migrating cells that were lost in cells knocked down for MAL2 expression (MAL2 KD). (**B**) Uninfected cells and cells knocked down for MAL2 at the migrating edge were double-labeled for MAL2 (with anti-FLAG antibodies) and actin (with phalloidin). Arrowheads in panels a and b indicate long, MAL2- and actin-positive protrusions present in uninfected migrating cells whereas arrowheads in c and d point out the smooth surfaces in migrating cells knocked down for MAL2 expression. (**C**) Cells from Part B were scored for the presence of protrusions. Values represent the number of MAL2 KD cells with protrusions relative to uninfected cells. (**D**) Cells knocked down for MAL2 expression were immunolabeled for actin with phalloidin. Arrowheads mark dorsal ruffle-like structures. (**E**) Uninfected and MAL2 overexpressing WIF-B cells present at the edge of a scratch were labeled for actin with phalloidin. Arrowheads mark long protrusions present at the cell surface. (**F**) Hep3B cells overexpressing MAL2 were treated for 4 h with 50 µg/mL cycloheximide and immunolabeled for MAL2 with anti-FLAG antibodies. Arrowheads point to MAL2 labeling present at the ends of the long protrusions. Bar = 10 µm.

When micrographs of migrating cells at the leading edge were enlarged (from boxed areas indicated in Figure 3B), we noticed that the numerous cell surface protrusions in control Hep3B cells (marked by arrowheads) were absent in cells knocked down for MAL2 expression (Figure 4A). Similar results were observed when cells were monitored using indirect immunofluorescence at higher magnification. In control Hep3B cells, many filopodia-like cell surface protrusions were present in cells at the leading edge with endogenous MAL2 detected in the tips (Figure 4B(a,b)). In contrast, cells knocked down for MAL2 expression had many fewer protrusions and displayed a more cuboidal phenotype (Figure 4B(c,d)). When quantitated, we found that MAL2 knockdown cells had 70% fewer protrusions than control Hep3B cells (Figure 4C). We also noticed circular, dorsal ruffle-like structures in MAL2 knockdown cells (an example is shown in Figure 4D) further implying enhanced invasiveness. To further determine whether MAL2 expression correlates with filopodia-like protrusion formation, we went back to the WIF-B cells. As shown in Figure 4E, when imaged at the leading edge of the scratch, both control cells (with endogenous MAL2 expression) and cells overexpressing wild type MAL2 exhibited protrusions. For comparison, we determined that Hep3B cells overexpressing wild type MAL2 had no further protrusion formation than in uninfected cells (Figure 4F). Together these results indicate that MAL2 protein expression promotes protrusion formation, yet impairs cell migration.

### 2.5. MAL2 Expression in Clone 9 Cells Induced Protrusion Formation, yet Impaired Cell Migration and Invasion

**Figure 5:**
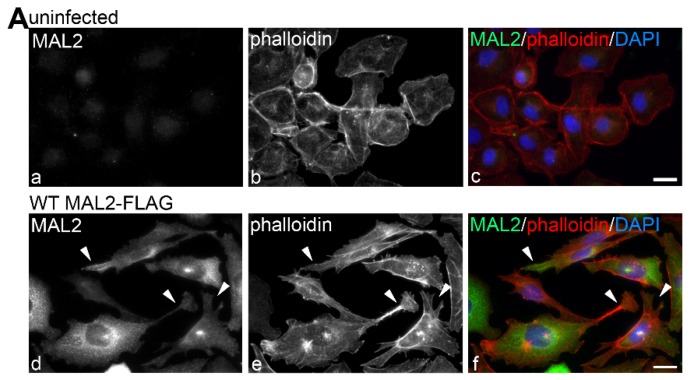

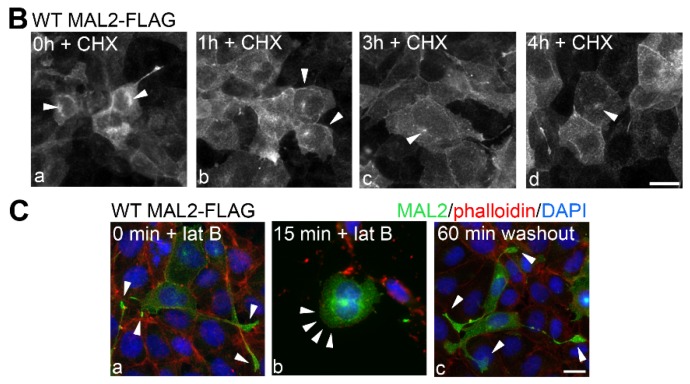
MAL2 expression in Clone 9 cells is associated with actin-based protrusion formation. (**A**) Uninfected Clone 9 cells and Clone 9 cells expressing wild type (WT) MAL2 were immunolabled for MAL2 (with anti-FLAG antibodies) and actin (with phalloidin). Nuclei were also stained with DAPI and merged images are shown in panels c and f. Arrowheads are marking actin-based protrusions in MAL2-expressing cells. (**B**) Clone 9 cells expressing FLAG-tagged wild type MAL2 were treated 50 µg/mL of cycloheximide (CHX) for up to 4 h as indicated and immunolabeled for MAL2 with anti-FLAG antibodies. Arrowheads indicate MAL2 localization at the Golgi (a), at the cell surface (b) and in the apical compartment (c,d). (**C**) Clone 9 cells expressing FLAG-tagged wild type MAL2 were immunolabeled for MAL2 (with anti-FLAG antibodies), actin (with phalloidin) and nuclei (with DAPI) after treatment with 5 μM latrunculin B (lat B) for 0 or 15 min or after washout for 60 min. Arrowheads in panels a and c mark long, actin-based protrusions in untreated cells and cells after latrunculin B washout. In panel b, arrowheads point to the smooth cell surfaces with no actin-based protrusion in cells treated for 15 min with latrunculin B. Bar = 10 µm.

To further confirm that MAL2 expression promotes actin-based protrusion formation, yet impairs cell migration, we expressed wild type MAL2 in the hepatoma-derived Clone 9 cells. Importantly, these cells lack endogenous MAL2 expression. As described above, Clone 9 cells are characterized by a cuboidal morphology with few cell surface protrusions (Figure 5A(a–c)). In contrast, in cells overexpressing MAL2, the cuboidal morphology was lost and more elongated cells were observed with long, cell surface filopodia-like, actin-based protrusions (Figure 5A(d–f)). As for both Hep3B and WIF-B cells, MAL2 expression was observed at the protrusion tips (Figure 5A(d–f)). To determine MAL2’s steady state distributions in Clone 9 cells, we treated cells with 50 µg/mL of cycloheximide (CHX) for up to 4 h. As observed for WIF-B cells, MAL2 distributions before cycloheximide treatment were observed throughout the biosynthetic pipeline (arrowheads mark the Golgi) and at the cell surface (Figure 5B(a)). In cells treated for 1 h, the diffuse MAL2 ER labeling and discrete Golgi labeling were diminished with a reciprocal increase in MAL2 detection at the cell surface (Figure 5B(b)). After 3 h, MAL2 surface labeling was lost with increased staining observed in the apical compartment, a structure that labels exclusively for apical membrane proteins in nonpolarized cells as we and other have previously extensively described (Figure 5B(c)) [26]. After 4 h, labeling at the “apical compartment” and cell surface were observed consistent with the recycling reported for apical residents between these two compartments in nonpolarized cells also as we have previously described (Figure 5B(d)) [26,27,28].

To confirm that the MAL2-induced protrusions are indeed actin-based structures, we treated cells with the actin depolymerizing agent, latrunculin B. In cells treated for only 15 min with 5 μM latrunculin B (lat B), all cell surface protrusions were lost (Figure 5C(b)). 

After 60 min of washout, the protrusions were reformed and cell morphology was restored (Figure 5C(c)) confirming the protrusions are actin-based. This further implies that MAL2 may interact with actin and/or actin-associated proteins to promote protrusion formation (see below).

**Figure 6:**
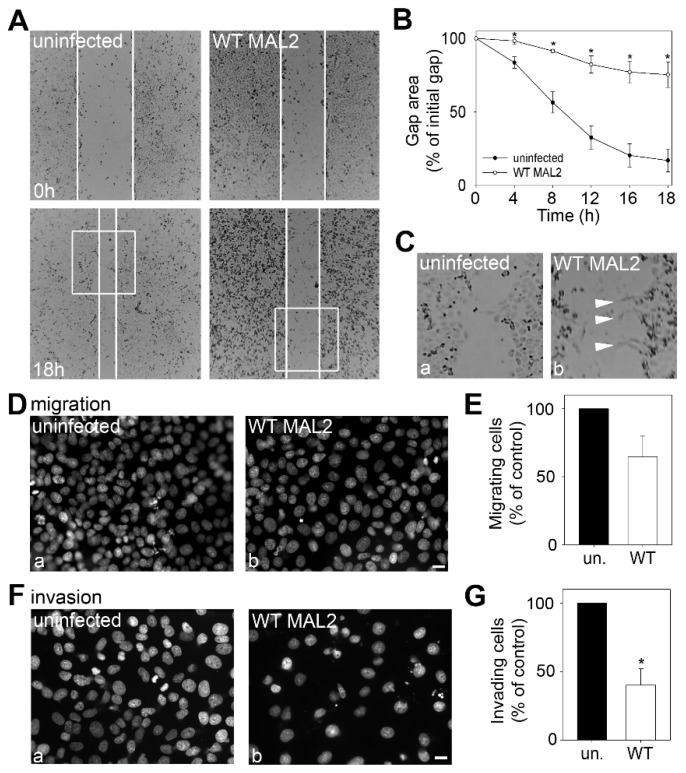
MAL2 expression in Clone 9 cells is associated with actin-based protrusion formation, yet impaired migration. (**A**) Monolayers of uninfected Clone 9 cells or cells expressing wild type (WT) MAL2 were scratched and wound healing was imaged at 0 h (upper panels) and 18 h (lower panels). White lines indicate the edges of the gap. (**B**) The gap area remaining after 18 h of recovery was determined relative to the area of the initial gap after 0, 4 (*p* ≤ 0.033), 8 (*p* ≤ 0.008), 12 (*p* ≤ 0.008), 16 (*p* ≤ 0.006) and 18 h (*p* ≤ 0.008) after recovery. Values represent the mean ± SEM determined from at least three independent experiments. (**C**) The boxed areas indicated in Part A were enlarged. Arrowheads point to long surface protrusions in MAL2-expressing cells present at the edge of the scratch. (**D**–**G**) Uninfected cells or cells expressing wild type MAL2 were seeded in serum-free medium onto filters in Boyden chambers in the absence (D,E) or presence of matrigel (F,G). After 24 h, the nuclei of cells that had penetrated to the underside of the filter were labeled with DAPI and visualized (D,F). In (E,G), the numbers of MAL2-expressing cells that had penetrated the filter were determined relative to the numbers of uninfected cells and plotted as the percent of control (uninfected). Values represent the mean ± SEM from at least three independent experiments. The difference in invasion was significant (*p* ≤ 0.05).

In scratch assays, Clone 9 cells (with no MAL2 expression) exhibited robust migration with near complete wound closure observed 18 h after the scratch (Figure 6A, bottom left panel). In contrast, wild type MAL2 expression led to visibly impaired migration (Figure 6A, bottom right panel). When quantitated, ~80% of the wound was closed after 18 h by uninfected Clone 9 cells whereas in cells expressing MAL2, only ~20% of the wound was closed (Figure 6B). This latter value is remarkably consistent with the rate of wound closure observed for Hep3B cells (that endogenously express MAL2). Also as for Hep3B cells, Clone 9 cells expressing wild type MAL2 at the leading edge of the scratch displayed abundant cell surface protrusions (marked with arrowheads) (Figure 6C(b)). We also examined whether MAL2 expression impaired cell migration and invasion using Boyden chamber assays. Consistent with results from the scratch assay, cells expressing wild type MAL2 displayed decreased migration through filters in Boyden chambers (Figure 6D). When quantitated, 30% fewer MAL2-expressing cells had traversed the filter (Figure 6E). Invasion across matrigel-coated filters was impaired to an even greater extent by MAL2 expression (Figure 6F) with up to 60% fewer cells recovered from the underside of the filter (Figure 6G).

**Figure 7:**
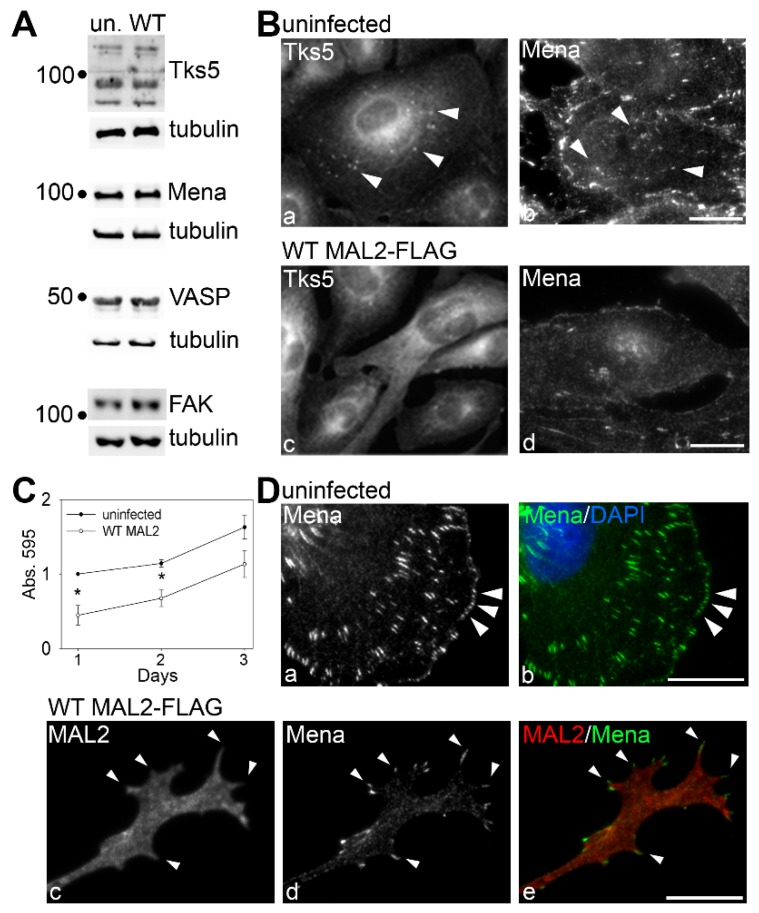
MAL2 overexpression disrupts invadopodia formation. (**A**) Lysates from uninfected Clone 9 cells and Clone 9 cells expressing wild type (WT) MAL2 were immunoblotted for Tks5, Mena, VASP, FAK and tubulin (as a loading control) as indicated. Molecular weight standards are indicated on the left in kDa. (**B**) Uninfected Clone 9 cells and Clone 9 cells expressing wild type MAL2 were immunolabeled for Tks5 or Mena as indicated. Arrowheads in panels a and b mark Tks5 and Mena present in juxta-nuclear invadopodia that are absent in cells expressing MAL2. (**C**) Uninfected and MAL2-expressing cells were seeded onto 96-well plates at 4000 cells/well and relative cell numbers monitored after 1 (*p* ≤ 0.006), 2 (*p* ≤ 0.006) or 3 days. Values represent the mean ± SEM from at least three independent experiments each performed in triplicate. (**D**) Uninfected Clone 9 cells were labeled for Mena. Arrowheads mark Mena present at the surface in patches. (**E**) Clone 9 cells expressing wild type MAL2 were immunolabeled for Mena and MAL2 as indicated. Arrowheads mark Mena present at the far distal tips of protrusions induced by MAL2 expression. Bar = 10 µm.

To confirm that MAL2 expression impaired the invasive phenotype, we analyzed Clone 9 cells for expression and distribution of selected invadopodia markers. Although no changes in tyrosine kinase substrate with five SH3 domains (Tks5), mammalian-enabled protein (Mena), vasodilator-stimulated phosphoprotein (VASP) or focal adhesion kinase (FAK) expression levels were observed on immunoblots of lysates from uninfected or MAL2-expressing Clone 9 cells (Figure 7A), decreased invadopodia were observed morphologically. In uninfected cells, both Tks5 and Mena localized to juxta-nuclear puncta characteristic of invadopodia labeling (Figure 7B(a,b)). In contrast, these puncta were lost in MAL2-expressing cells (Figure 7B(c,d)). Interestingly, Mena localized to the extreme distal tips of the MAL2-induced protrusions just past regions of the tips positive for MAL2 labeling (Figure 7D(c–e)) consistent with them being filopodia-like structures. We also measured relative cell numbers in uninfected and MAL2-overexpressing cells up to three days after plating. As shown in Figure 7C, cells expressing MAL2 displayed a modest, but significant decrease in absorbance levels after 1 (*p* ≤ 0.006), 2 (*p* ≤ 0.006) or 3 days suggesting decreased proliferation. Together these data are consistent with MAL2 function as a tumor suppressor.

### 2.6. MAL2 Tumor Suppressor Activity is Mediated by a Putative EVH1 Recognition Motif

**Figure 8:**
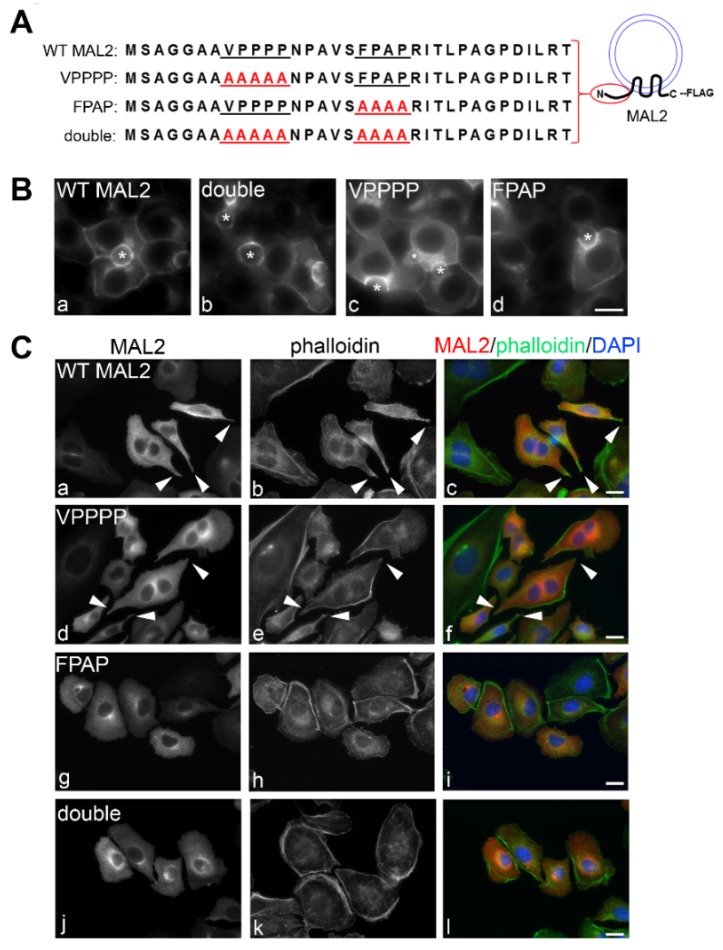
Mutations in a putative EVH1 recognition site decrease protrusion formation. (**A**) A diagram of the cytosolic, N-terminal amino acid sequence of MAL2 containing is shown. The two putative proline-rich EVH1 recognition sites are underlined and the single and double mutations are indicated in red. All constructs were FLAG-tagged at the C-terminus. (**B**) WIF-B cells expressing the wild type (WT) and mutant MAL2 proteins were immunolabeled for MAL2 with anti-FLAG antibodies. Asterisks mark bile canaliculi. (**C**) Clone 9 cells expressing wild type (WT) and the mutant MAL2 proteins were double labeled for MAL2 (with anti-FLAG antibodies) and actin (with phalloidin). Merged images are shown in c, f, i and l with DAPI labeling of nuclei. Arrowheads in a–f indicate surface protrusions in cells expressing wild type or VPPPP mutant MAL2. Bar = 10 µm.

The MAL2 cytoplasmically-oriented N-terminal domain is the most divergent among MAL family members. Only MAL2 encodes VPPPP and FPAP sequences (underlined) that resemble the F/L/W/YPPPP recognition sites for EVH1 (enabled, VASP, homology1) motifs present in Ena/VASP proteins [29] (Figure 8A). Because MAL2 expression promoted actin-based protrusion formation at the likely expense of invadopodia formation, we generated recombinant adenoviruses expressing FLAG-tagged MAL2 mutants with each of these sites mutated to alanines alone (VPPPP and FPAP) or together (double) (indicated in red) (Figure 8A). A FLAG epitope was added to the C-terminus for each mutant.

**Figure 9:**
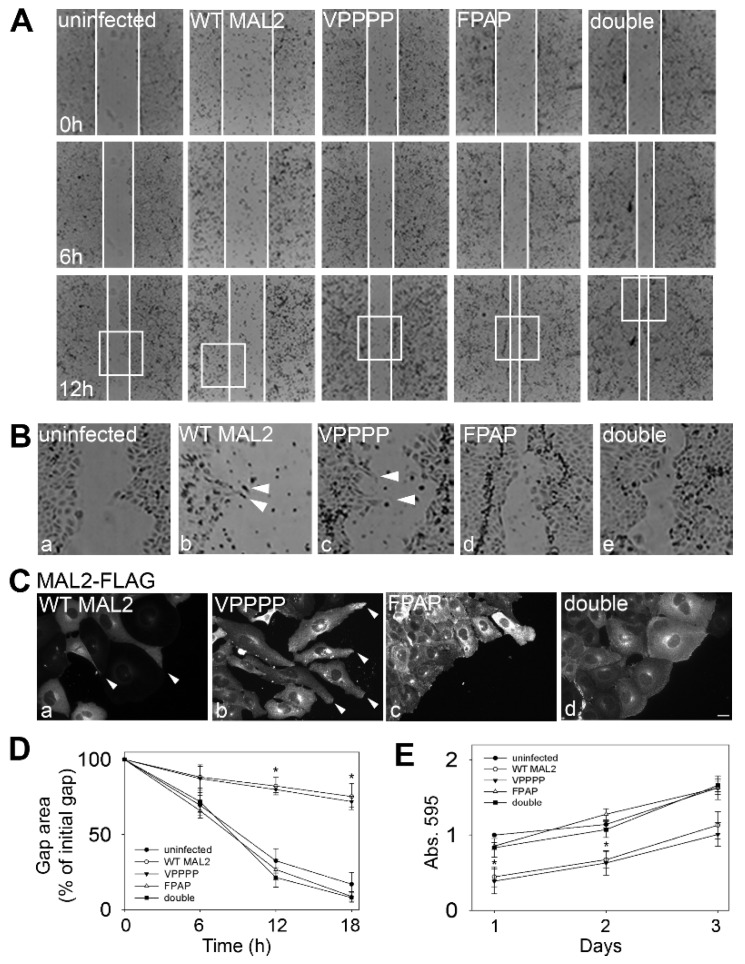
The FPAP motif mediates MAL2-induced protrusion formation and impaired cell migration. (**A**) Monolayers of infected cells or cells expressing wild type (WT) or mutant MAL2 as indicated were scratched and wound healing was imaged 0 h (upper panels), 6 h (middle panels) and 12 h (lower panels) post-scratch. White lines indicate the edges of the gap. (**B**) The boxed areas in Part A were enlarged. Arrowheads in panels c and d indicate protrusions in cells expressing wild type or the VPPPP mutant MAL2 at the migrating edge. (**C**) Cells at the migrating edge were immunolabeled for MAL2 (with anti-FLAG antibodies). The Arrowheads in panels a and b indicate protrusions in cells expressing wild type or the VPPPP mutant MAL2 at the migrating edge. Bar = 10 µm. (**D**) The gap area remaining after 0, 6, 12 (*p* ≤ 0.008 for WT and ≤ 0.005 for VPPPP) and 18 h (*p* ≤ 0.008 for WT and ≤ 0.005 for VPPPP) of recovery was determined relative to the area of the initial gap in cells expressing wild type and mutant MAL2 as indicated. Values represent the mean ± SEM from at least five independent experiments. (**E**) Uninfected or MAL2-expressing cells were seeded onto 96-well plates at 4000 cells/well and relative cell numbers monitored after 1 (*p* ≤ 0.006 for WT and ≤0.01 for VPPPP), 2 (*p* ≤ 0.008 for WT and ≤0.024 for VPPPP) or 3 days. Values represent the mean ± SEM from at least three independent experiments each performed in triplicate.

To first confirm that the MAL2 mutants fold properly, we examined their distributions in polarized WIF-B cells. As shown in Figure 8B, all three mutants showed identical distributions to that of overexpressed wild type MAL2 (see Figure 2D for comparison). As for wild type, the mutants were detected throughout the biosynthetic pathway and at both surface domains. We next examined the morphology of Clone 9 cells expressing the three MAL2 mutants. Unlike Clone 9 cells expressing wild type MAL2, no cell surface protrusions were observed in cells expressing MAL2 with both putative EVH1 motifs mutated (double) (compare Figure 8C(a–c) with Figure 8C(j–l)). The wild type phenotype was also lost in cells expressing the FPAP mutant MAL2 (Figure 8C(g–i)), but not the VPPPP mutant (Figure 8C(d–f)) indicating that the FPAP motif selectively mediates MAL2-induced protrusion formation. As predicted, cells with enhanced surface protrusion formation (expressing wild type or the VPPPP mutant) displayed decreased motility in scratch assays than those without protrusions (expressing the FPAP or double mutant MAL2) (Figure 9A). When quantitated, cells expressing the FPAP or double mutant MAL2 had overlapping rates of gap closure as uninfected cells (~80–90% closure after 18h) (Figure 9D) compared to only 20% observed in cells expressing wild type or VPPPP mutant MAL2 (Figure 9D). 

When Clone 9 cells at the leading edge (Figure 9B) were imaged by indirect immunofluorescence similar changes in cell morphology were apparent with protrusion formation observed in cells expressing wild type or VPPPP mutant MAL2 that was absent in cells expressing the FPAP or double MAL2 mutants (Figure 9C). When cells were scored for cell surface protrusions, more than half of cells expressing wild type (69%) or the VPPPP mutant MAL2 (56%) were positive for actin-based protrusions while significantly fewer uninfected cells (1%) or cells expressing the FPAP (7%) or double MAL2 mutants (16%) displayed protrusions. We also we monitored relative cell numbers in control and infected Clone 9 cells. Modest, but significant decreases in absorbance reading after 1 (*p* ≤ 0.006 for WT and ≤ 0.01 for VPPPP), 2 (*p* ≤ 0.008 for WT and ≤ 0.024 for VPPPP) or 3 days were observed suggesting that decreased proliferation correlated with protrusion formation.

**Figure 10:**
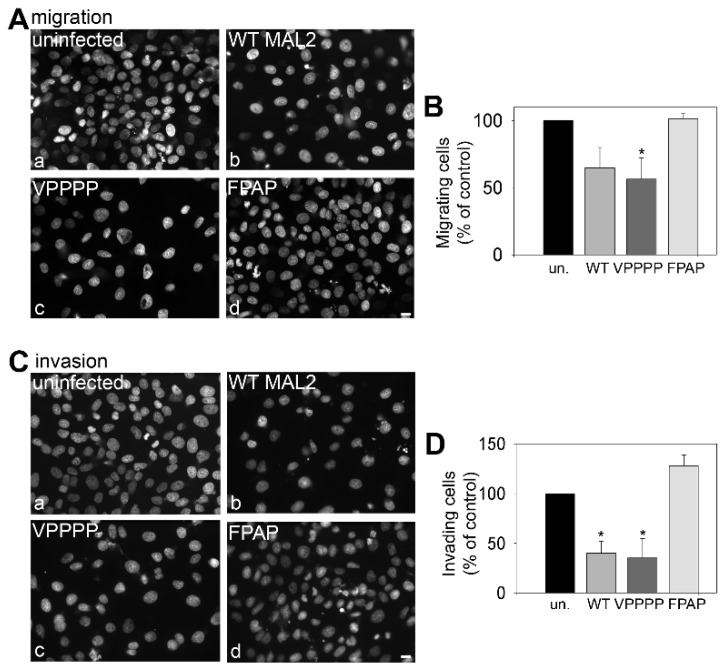
The FPAP motif mediates MAL2-induced protrusion formation and impaired cell migration. (**A**–**D**) Uninfected cells or cells expressing wild type of mutant MAL2 were seeded in serum-free medium onto filters in Boyden chambers in the absence (A,B) or presence of matrigel (C,D) cells. After 24 h, the nuclei of cells that had penetrated to the underside of the filter were labeled with DAPI and visualized (A,C). In (B,D), the numbers of MAL2-expressing cells that had penetrated the filter were determined relative to the numbers of uninfected cells and plotted as the percent of control (uninfected). In (B), * *p* ≤ 0.049 and in (D), *p* ≤ 0.007 for WT and ≤0.029 for VPPPP. Bar = 10 µm.

Boyden chamber migration and invasion assays were fully consistent with these results. Clone 9 cells expressing wild type or the VPPPP mutant MAL2 displayed significantly reduced migration (Figure 10A) and invasion (Figure 10C) than observed for uninfected cells or those expressing the mutant FPAP MAL2. When quantitated, cells expressing wild type or the VPPPP mutant MAL2 displayed a significant decrease in migration (by ~40%) and invasion (by ~60%) with no changes in migration observed in uninfected cells or cells expressing the FPAP mutant MAL2 (Figure 10B,D). Together these data indicate that the FPAP (but not VPPPP) motif mediates MAL2 tumor suppressor activity.

**Figure 11:**
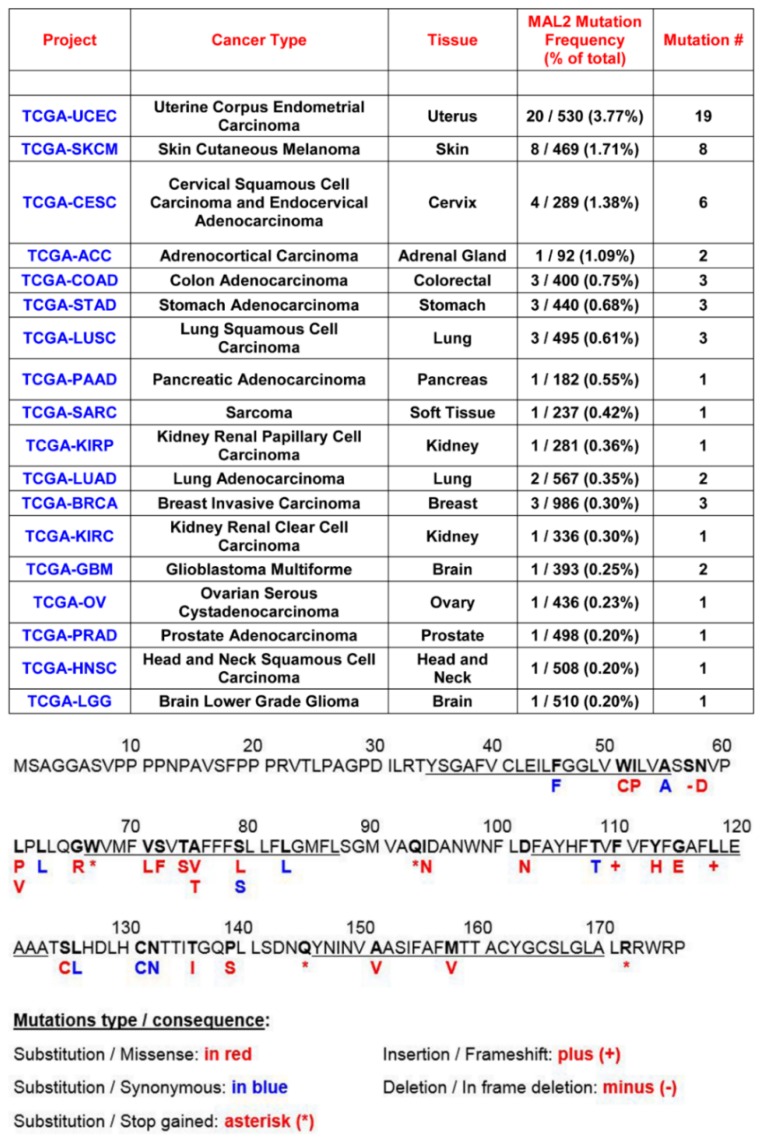
MAL2 has a low somatic mutation frequency in human cancers. MAL2 mutation rates for 18 different human cancer types were determined from data compiled by the NCI Genomic Data Commons. Below, the specific patient mutations (and consequences) in the MAL2 amino acid sequence are indicated in blue and red. MAL2 transmembrane domains are underlined.

Based on these results, one possible explanation to reconcile MAL2’s function as a tumor suppressor with its upregulated expression and link to bad prognosis is that overexpressed MAL2 harbors mutations in the FPAP motif thereby abolishing its tumor suppressor activities. However, results from the NCI Genomic Data Commons (GDC) [30] indicate that no such MAL2 mutations have been observed in almost 8000 patients representing 18 different cancer types (Figure 11). In fact, MAL2 exhibits a strikingly low overall somatic mutation rate with mutations observed in an average of 0.75% of the 7689 cases analyzed (Figure 11). Of those few mutations, none fall within the divergent MAL2 N-terminal domain, but instead map mainly to the transmembrane domains. Thus, MAL2 mutation patterns and/or rates cannot resolve this paradox. However, based on our reading of the primary literature, we have formulated a possible explanation to resolve this paradox (see Discussion).

## 3. Discussion

Blinded and independent analysis of human HCC, CC and RCC tissue revealed that MAL2 protein expression levels were surprisingly, significantly decreased in malignant lesions more consistent with MAL2 function as a tumor suppressor. Using three hepatic cell model systems, we determined that MAL2 protein overexpression was anti-oncogenic and led to decreased cell migration, invasion and viability (albeit to a lesser extent). MAL2 expression also led to the formation of filopodia-like actin-based protrusions with a reciprocal decrease in invadopodia and/or lamellipodia formation. From mutational analysis, we determined that the putative EVH1 recognition motif (FPAP) in the cytoplasmically-oriented MAL2 N-terminal domain mediates protrusion formation and decreased migration and invasion.

### 3.1. MAL2-Mediated Actin Remodeling and Tumorigenesis

The mutational analysis presented here suggests that MAL2 coordinates with actin binding proteins to regulate protein sorting in both polarized cells and that in the non-polarized cell context, this leads to protrusion formation. Many lines of evidence support a link between MAL2 function in vesicle docking, budding and fusion and the actin network. We determined that MAL2 redistributes from the apical membrane to sub-apical puncta upon depolymerization of the cortical actin web [21]. Inverted formin 2 (INF2) was identified as a MAL2 binding partner in yeast two hybrid analysis [31]. Studies in polarized HepG2 cells suggested that INF2-mediated actin remodeling is likely required for MAL2’s role in transcytosis. More recently, we determined that MAL2 may coordinate with its known binding partner, serine threonine kinase 16, and the actin binding protein, WD repeat-containing protein 1, to regulate secretory vesicle docking and fusion at the basolateral surface [25].

Based on our studies here, we further propose that MAL2-mediated actin remodeling may also be mediated by Ena/VASP proteins that drive barbed-end actin filament assembly, and that in the nonpolarized cell context, this drives filopodia-like protrusion formation. We further propose that this protrusion formation may antagonize the oncogenic phenotype. In the absence of MAL2, actin, actin-binding proteins and other proteins are directed to sites of invadopodia formation thereby promoting the oncogenic phenotype. MAL2 expression redirects these components away from the ventral invadopodia sites to the lateral edges. This is consistent with Mena redistribution from ventral puncta to the tips of the MAL2-induced protrusions. However, an open question is why these protrusions are associated with decreased migration and proliferation. One possibility is that MAL2 is inducing more adhesive filopodia formation at the expense of more Arp2/3-rich lamellipodia associated with more rapid migration [32]. Nonetheless, as long as MAL2 expression is maintained, full oncogenic transformation may be prevented. A possibility we are actively investigating.

### 3.2. A MAL2 Paradox

**Figure 12:**
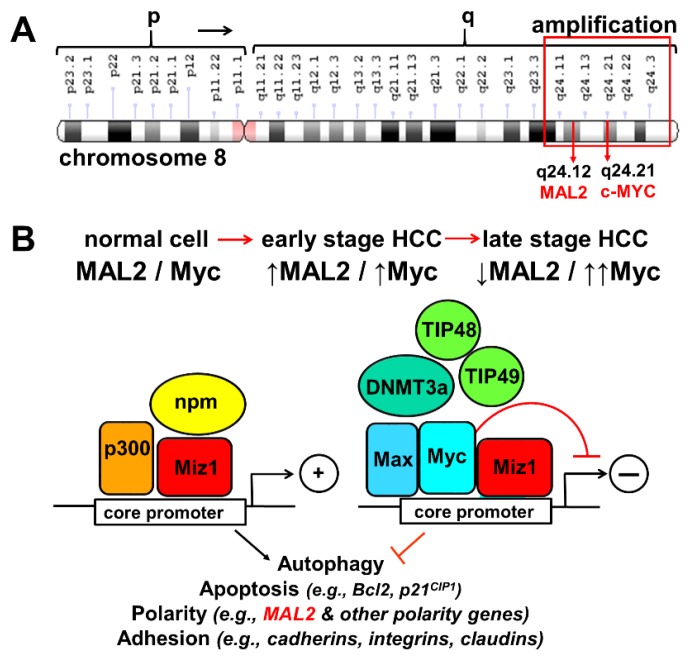
Explaining the MAL2 paradox. (**A**) *MAL2* and *c-Myc* are on chromosome 8q24, a region frequently amplified in epithelial-derived cancers. (**B**), In normal cells with low c-Myc (left), Miz1 binds a core promoter with p300 and nucleophosmin (npm) and activates transcription of genes involved in tumor suppression (including MAL2). Upon 8q24 amplification in early HCC, both MAL2 and myc levels are increased. As transformation progresses, Myc levels are increased (right), Myc-Max dimers bind Miz1, displace p300 and npm, recruit a methyl transferase and other factors thereby repressing Miz1 transcriptional activity and lead to decreased MAL2 expression and enhanced transformation.

This work was motivated by the finding that MAL2 is upregulated in a host of human epithelial-derived cancers at least at the transcript level. However, we determined that MAL2 protein expression levels are decreased in HCC, CC and RCC, displays an extremely low somatic mutation rate in human cancers and its function is more consistent with it being a tumor suppressor leaving us with a paradox. How can MAL2’s function as a tumor suppressor be reconciled with its upregulated expression and link to bad prognosis in human cancers? The amplification of chromosomal 8q24 has been documented in many epithelial cell-derived cancers and is particularly associated with progression of HCC and CC [33,34]. Although *MAL2* resides in this amplified region (at 8q24.12), another gene on chromosome 8q24 has garnered much more attention, *c-MYC* (at 8q24.21) [33,34] (Figure 12). Myc dysregulation and overexpression is found in virtually all human epithelial-derived cancers and is often associated with poor prognosis [35,36]. It is widely appreciated that Myc overexpression leads to the transcription of countless genes that promote transformation and metastasis [35,36]. Further reinforcing this progression, Myc also represses other transcription factors that activate genes involved in tumor suppression, including Myc-interacting zinc finger protein-1, (Miz1, also known as Pias2 and ZBTB17) [35,37]. In normal cells, Myc dimerizes with Max while Miz1, at core promoter sequences, promotes expression of genes involved in cell-cell and cell-matrix adhesion, autophagy, apoptosis and polarity (including MAL2). In cancer cells with high c-Myc expression, Myc-Max complexes are additionally recruited to Miz1 at its promoter, displacing other factors thereby repressing Miz1 transcriptional activity leading to decreased apoptosis, cell adhesion, autophagy, and polarity [35,36,37].

To resolve the “MAL2 Paradox”, we propose the following scenario. In human HCC and CC, chromosome 8q24 is amplified leading to enhanced expression of both MAL2 and c-Myc. As the cancer progresses, Myc expression is further enhanced leading to Miz1 transcriptional repression and loss of Miz1-specific target expression—including MAL2. Thus, the simple prediction is that enhanced MAL2 expression is associated with earlier stages of cancer progression and its expression is repressed during later stages and in metastases as Myc protein expression increases. We are systematically determining the expression patterns of MAL2, c-Myc and Miz1 (and other selected targets) in HCC and CC with increasing grades of tumors to establish this temporal relationship (manuscript in preparation).

## 4. Materials and Methods

### 4.1. Reagents and Antibodies

F12 and F12 (Coon’s modification) medium, latrunculin B, cycloheximide (Cat. No. C7698-1G) was from Sigma-Aldrich (St. Louis, MO, USA). Fetal bovine serum (FBS) and newborn calf serum were from Gemini Bio-Products (Woodland, CA, USA). Anti-α-tubulin mouse monoclonal antibodies (DM1A, Cat. No. T9026, lot no. 052M4837), anti-FLAG mouse monoclonal antibodies, were from Sigma-Aldrich. Rabbit polyclonal antibodies against full length human MAL2 (Cat. No. ab75347), and VASP (Cat. No. ab47461) were from AbCam (San Francisco, CA, USA). Rabbit polyclonal antibodies against Tks5 (Fish (M-300)) were from Santa Cruz (Dallas, TX, USA) (Cat. sc-30122). Rabbit polyclonal antibodies against Mena (Cat. No. NBP1-87914) were from NOVUS (Littleton, CO, USA). Rabbit polyclonal antibodies against rat MAL2 were generated and affinity purified as described previously [4]. Monoclonal antibodies against rat zonula occludens-1 (ZO-1) were generously provided by Dr. Ann Hubbard (Johns Hopkins University School of Medicine, Baltimore, MD, USA) and are now part of our lab stocks. Alexa488 and -568-conjugated secondary antibodies were purchased from Invitrogen Life Technologies (Carlsbad, CA, USA) and HRP-conjugated secondary antibodies were from Sigma-Aldrich.

### 4.2. Human Tissue Immunohistochemistry (IHC) Staining

It should be noted that this evaluation was conducted and interpreted in a blinded fashion, with the clinical team having no knowledge of the results of other experimental findings. Paraffin-embedded tissues were cut into ~4 µm sections and pre-treated with heat-induced epitope retrieval (HIER) and diluted Envision FLEX Target Retrieval Solution at pH 6.0 (50×) (K8004) for Ki-67 (IR626, Clonal MIB-1, Agilent, Santa Clara, CA, USA) and MAL2 (Cat. No. ab75347, Abcam). Deparaffinization, rehydration and epitope retrieval were performed in DAKO PT Link (PT100/PT101) preheated to 85 °C followed by epitope retrieval at 97 °C for 20 min. Samples were cooled at 65 °C. Sections were soaked in diluted Envision FLEX Wash Buffer (20×) (K8007) for 5 min. Staining was performed using an Autostainer Link 48 DAKO. Sections were blocked with FLEX Peroxidase Blocking Solution (SM801) for 5 min. Anti-Ki-67 (ready-to-use) and anti-MAL2 (1:100) antibodies were separately added to different slides for 20 min followed by FLEX Mouse Linker (SM804) for 15 min, FLEX HRP (SM802) for 20 min, FLEX DAB with Substrate-Chromogen (SM803) for 10 min and FLEX hematoxylin (SM806) for 5 min. Between the different applications, the sections were washed for 5 min with diluted Envision FLEX Wash Buffer (20×) (K8007).

### 4.3. Densitometry and Statistical Analysis

Densitometric comparison of immunoreactive bands was performed using ImageJ 1.47v software (National Institutes of Health, Bethesda, MD, USA). In general, images were inverted and averaged pixel intensity x area for each band was determined, blank-corrected and normalized to an appropriate loading control (see legends for details). Results were expressed as the mean ± SEM from at least three independent experiments. Comparisons between experimental groups were made using the Student’s two-tailed *t* test for paired data. *P*-values ≤ 0.05 were considered statistically significant.

### 4.4. Study Approval

Immunohistochemical staining of the HCC, RCC, and CC tissue sections was strictly performed for research and investigational purposes. The studies presented here are considered secondary research for which consent is not required. The samples were retrieved from a large bank of patient biospecimens that were previously collected. The information provided about the biospecimens was recorded by the original investigator in such a manner that the identity of the human subjects cannot readily be ascertained directly or through identifiers linked to the subjects. Also, the current investigators will not contact the subjects and will not re-identify subjects. The findings will not modify the patients’ initial diagnoses or alter their current course of treatment. For these reasons Institutional Review Board exemption was granted from MedStar Georgetown University Hospital (IRB exemption number: 2018-0022) and the Catholic University of America (IRB exemption number 19-0031). The authors would like to thank the patients and their families for providing the tissue samples that were provided with informed consent at the time of collection.

### 4.5. Cell Culture

WIF-B cells were grown in a humidified 7% CO_2_ incubator at 37 °C as described [38]. Briefly, cells were cultured in F12 (Coon’s modification) medium supplemented with 5% fetal bovine serum (FBS), 10 μM hypoxanthine, 40 nM aminoterpin and 1.6 μM thymidine, pH 7.0. Clone 9 cells were cultured in F-12 (Coon’s modification) medium supplemented with 10% new born calf serum. Hep3B cells were cultured in Dulbecco’s Modified Eagle Medium (DMEM) supplemented with 10% FBS. Both Hep3B and Clone 9 cell lines were grown in a humidified 5% CO_2_ incubator at 37 °C. WIF-B cells were seeded onto glass coverslips at 1.3 × 10^4^ cells/cm^2^ and cultured for 8–12 days until they reached maximal density and polarity. Clone 9 and Hep3B cells were seeded onto glass coverslips in 6-well dishes at 0.5–1.0 × 10^6^ cells/well and cultured for 1–2 days.

### 4.6. RNA Extraction and Semi-Quantitative RT-PCR

Total RNA was extracted and purified with the RNeasy Mini Kit (Qiagen, Germantown, MD, USA) according to the manufacturer’s instructions. Reverse-transcription (RT)-PCR was performed with 1 µg RNA template using OneStep RT-PCR kit (Qiagen) also according to the manufacturer’s instructions. PCR was performed using the following parameters: 30 min at 50 °C, 15 min at 95 °C, 30 cycles of 30 s at 94 °C, 30 s at 55 °C, 1 min at 72 °C, and 10 min at 72 °C. Primer sequences used for amplification were as follows: rat MAL2, forward 5′-ATGTCGGCCGGCGGAG-3′, reverse 5′-TTACGGTCGCCATCTTC GCA-3′; human MAL2, forward 5′-CATCCTGCGGACCTACTCG-3′, reverse 5′-CACGGACACAAA CATGACC-3′; rat α-tubulin, forward 5′-CCAGGGCTTCTTGGTTTTCC-3′, reverse 5′-CGCTCAATG TCGAGGTTTCT-3′; human α-tubulin, forward 5′-ACACTGTGGTTGAGCCCTACA-3′, reverse 5′-GCTTGAGGGTGCGGAAA-3′. PCR products were analyzed on 1% agarose gel containing ethidium bromide.

### 4.7. Virus Production and Infection

Full-length rat MAL2 in a pCMV-SPORT6 vector was purchased from Life Technologies (Carlsbad, CA, USA). Recombinant adenoviruses encoding C-terminally FLAG epitope-tagged full-length wild-type, mutated VPPPP-to-AAAAA, mutated FPAP-to-AAAA, double mutated VPPPP/FPAP-to-AAAAA/AAAA MAL2 mutants and antisense MAL2 were generated using the ViraPower Adenoviral Expression System (Life Technologies), according to the manufacturer’s instructions and as described. All mutations were made using the Q5 Site-Directed Mutagenesis kit (New England Biolabs, Ipswich, MA, USA) according to the manufacturer’s instructions and verified by plasmid sequencing (Retrogen, San Diego, CA, USA). WIF-B, Clone 9 and or Hep3B cells were infected with recombinant adenovirus particles for 60 min at 37 °C as described [39]. Complete medium was added to the cells and they were incubated an additional 16–24 h to allow for protein expression.

### 4.8. Immunofluorescence Microscopy

Control and infected cells were grown on glass coverslips were either fixed on ice with chilled PBS containing 4% paraformaldehyde (PFA) for 1 min and permeabilized with ice-cold methanol for 10 min or fixed at room temperature with 3.7% PFA for 10 min and permeabilized for 5 min with 0.5% Triton X-100 in PBS. For phalloidin (Life Technologies, product A12379) staining, cells were permeabilized with 3.7% PFA for 15 min and permeabilized for 5 min with 0.1% Triton X-100/PBS. Cells were labeled for 1 h at RT with phalloidin diluted in 1% BSA/PBS to 1:20. Coverslips were mounted in DAPI-conjugated mounting media onto slides. Cells were processed for indirect immunofluorescence as previously described [26] Alexa 488- or 568-conjugated secondary antibodies were used at 100 nM. Labeled cells were visualized at RT by epifluorescence with an Olympus BX60 Fluorescence Microscope (OPELCO, Dulles, DC, USA) using an UPlanFl 60×/NA 1.3, phase 1, oil immersion objective. Images were taken with an HQ2 CoolSnap digital camera (Roper Scientific, Berlin, Germany) and Metamorph Imaging software (Molecular Devices, Sunnyvale, CA, USA). Adobe Photoshop (Adobe Systems Inc., Mountain View, CA, USA) was used to process images and to compile figures.

### 4.9. 2D Scratch Assays

Cells were seeded onto glass coverslips in tissue culture plates to confluency. Cell monolayers were wounded with a plastic pipette tip and phase-contrast images were captured using the CytoSMART live cell imaging system from Lonza (Basel, Switzerland) every 5 min for up to 24 h. Cell coverage and motility was measured using Lonza CytoSMART system software.

### 4.10. Boyden Chamber Migration and Invasion Assays

Migration and invasion assays were conducted in 24-well Transwell inserts (8 µm pore; Costar, Corning, NY, USA). For the migration assays, cells were trypsinized 24 h post-infection and resuspended in serum-free media. 4 × 10^4^ cells were seeded in 100 µL of serum-free media in the upper chamber of the Transwell insert. Five hundred µL of complete media was added to the lower chamber as a chemoattractant. For the invasion assays, cells were trypsinized 24 h post-infection and resuspended in serum-free media. 1 × 10^5^ cells/well were seeded in 100 µL of serum-free media in the upper chamber of the Transwell insert precoated with 50 µL matrigel (1:3 dilution, BD Biosciences, San Jose, CA, USA). 500 µL of complete media was added to the lower chamber as a chemoattractant. Cells were allowed to migrate and/or invade during an overnight incubation at 37 °C. Cells that did not migrate or invade were removed from the inside of the upper chamber with a cotton swab. Cells on the filter undersides were fixed in 4% paraformaldehyde for 10 min at room temperature, rinsed with 1× PBS and nuclei stained with DAPI-conjugated mounting media and mounted on glass slides. Stained cells were visualized by epifluorescence microscopy. Five randomly chosen fields were visualized using a 40× objective and cells counted. Data is presented as the percent of control migrating or invading cells.

### 4.11. Cell Proliferation Assay

Four thousand cells were seeded onto 96-well plates at the same 24 h post-infection in F12 (Coon’s modification) medium supplemented with 5% fetal bovine serum (FBS) for 3 days. Cell proliferation was determined using the MTT assay (Sigma-Aldrich) according to the manufacturer’s instructions.

## 5. Conclusions

Blinded and independent analysis of human HCC, CC and RCC tissue revealed that MAL2 protein expression levels were surprisingly, significantly decreased in malignant lesions more consistent with MAL2 function as a tumor suppressor. Using three hepatic cell model systems, we determined that MAL2 protein overexpression was anti-oncogenic and led to decreased cell migration, invasion and viability (to a lesser extent). MAL2 expression also led to the formation of filopodia-like actin-based protrusions with a reciprocal decrease in invadopodia and/or lamellipodia formation. From mutational analysis, we determined that the putative EVH1 recognition motif (FPAP) in the cytoplasmically-oriented MAL2 N-terminal domain mediates protrusion formation and decreased migration and invasion. To reconcile our findings of decreased MAL2 protein expression in human HCC, CC and RCC and its anti-oncogenic phenotypes with studies from others where increased MAL2 increased transcript levels were measured in a variety of human carcinoma types, we propose a transcriptional regulatory model for MAL2 transient overexpression.
